# Re-introduction of the Saker Falcon (*Falco cherrug*) in Bulgaria - preliminary results from the ongoing establishment phase by 2020

**DOI:** 10.3897/BDJ.9.e63729

**Published:** 2021-04-20

**Authors:** Ivanka Lazarova, Rusko Petrov, Yana Andonova, Ivaylo Klisurov, Andrew Dixon

**Affiliations:** 1 Trakia University, Stara Zagora, Bulgaria Trakia University Stara Zagora Bulgaria; 2 Green Balkans - Stara Zagora NGO, Stara Zagora, Bulgaria Green Balkans - Stara Zagora NGO Stara Zagora Bulgaria; 3 Reneco International Wildlife Consultants, Abu Dhabi, United Arab Emirates Reneco International Wildlife Consultants Abu Dhabi United Arab Emirates

**Keywords:** Saker Falcon, Falco cherrug, captive breeding, hacking, breeding performance, post-fledging dependence period

## Abstract

Considered extinct as breeding species in the early 2000s, the Saker Falcon was recovered when the first active nest from the new history of the species in Bulgaria was discovered in 2018, formed of two birds that were re-introduced back in 2015. Currently, there is only one confirmed wild breeding pair in the country - the male from 2015 with a female changed in 2020, released again as a part of the programme, in 2016. This is a report on the preliminary results and analysis of the ongoing establishment phase of the re-introduction of the Saker Falcon (*Falco cherrug*) in Bulgaria - first ever performed for this species in the country and globally. The period studied is 2015-2020. Following the re-introduction activities started in 2011, the current phase is defined by standardised methodology and a unified approach. Analysed and presented are methods for captive breeding and hacking, the breeding performance of the falcons, the number of released individuals, data from the post-fledging dependence period and a model of population growth.

## Introduction

The Saker Falcon (*Falco cherrug*) is a species of the Palearctic avifauna inhabiting plains and forest-steppes in the West and semi-desert montane plateaus and cliffs in the East (Ferguson-Lees and Christie 2001). Its nesting range includes territories in Central and Southern Europe and Asia as far east as China. The majority of the Central and Eastern European population is migratory and winters in the Mediterranean, the Near East and East Africa (Cramp and Simmons 1980). The global population is estimated between 6,100 - 14,900 (median 10,500) breeding pairs (Kovács et al. 2014), although data for a significant part of the species breeding range in Asia are missing (Dixon 2007, Dixon 2009).

At the end of the 19^th^ and beginning of the 20^th^ century, the Saker Falcon was a relatively numerous and widespread species in Bulgaria (Farman 1868). Not long after, the population started to decrease dramatically and, by the mid-20^th^ century, there were no records of confirmed breeding pairs within the country (Patev 1950). After 1970, a certain increase in the number and distribution of the species was observed, reaching 20-40 pairs by 1980 (Michev and Petrov 1985). Despite the efforts of several conservation organisations to preserve the population, it began to decline again and the last occupied nest was recorded in 2006. In the following years, a number of pairs and occupied territories were registered which indicated probable nesting; however, there were no records to confirm these suggestions (Iankov and Gradinarov 2012, Ragyov et al. 2014).

The main reason identified for the dramatic decline of the raptor populations in Bulgaria, including that of the Saker Falcon, was the significant habitat loss due to changes in land use - the transition from grazing to arable crops which led to diminishing of key food sources (Baumgart 1978, Lazarova and Balieva 2020). Amendments to the national legislation on hunting and animal protection through the years opened a gap which led to the widespread use of poisonous baits and the accumulation of pesticides in the food chain (Knaus et al. 2018, Lazarova and Balieva 2020) which, in turn, led to significant poisoning of birds of prey. In addition, a number of sources linked the decline of the population after 1985 to the increase in illegal trade of nest-poached chicks and eggs (Ragyov and Shishkova 2006, Ruskov et al. 2007). Other key factors related to the decrease in the Saker Falcon population, both globally and in Bulgaria, appeared to be power line electrocution, capture of individuals during migration, collision with artificial structures and vehicles and lack of suitable nesting places (Kovács et al. 2014, Iankov and Gradinarov 2012). As a result of this negative trend of rapid population decline due to the above-listed anthropogenic factors, the Saker Falcon was uplisted in the IUCN Red List as Endangered species (BirdLife International 2017). Any action against endangered species welfare in Bulgaria is treated as environmental or wildlife crime under the terms of the Biodiversity Act (2002). Furthermore, all acts of cruelty towards a vertebrate animal, including birds, fall within the Penal Code jurisdiction, resulting in fine or imprisonment (Kirov et al. 2019).

Despite the intensified conservation efforts, the implementation of European legislation for protection of wildlife and regulations for the registration and use of pesticides in Bulgaria since 2007 (Nikolova et al. 2015), a significant stabilisation of the Saker Falcon population was not observed. The endangered status and threats for wildlife species have further triggered the joint conservation efforts between NGOs and national authorities in the country (Nikolova 2010). In 2009, a team of international organisations conducted a feasibility study assessing the need for a re-introduction of the Saker Falcon in Bulgaria and the strategies needed to implement such a programme (Ragyov et al. 2009). Later studies developed a number of criteria for determining the most suitable locations for the restoration of the species in the region of central Stara Planina mountain. A number of preparatory pilot measures were implemented, related to delivery of food supplies to the species, together with activities for power lines insulation with the aim of ensuring optimal conditions for the Saker Falcon re-introduction (Ragyov et al. 2012) and re-introduction goals of forming 4-6 breeding pairs set up (Ragyov et al. 2010). In the period 2012-2014, ten captive-bred Saker Falcons were successfully released from adaptation aviaries (hacks) and tracked via satellite transmitters. From all hacked birds, tracked by satellite, which survived to their second summer when they could potentially breed, two showed regional and local-scale natal philopatry. This observation indicated that hacking can be used to restore breeding Saker Falcons to specific geographical locations (Dixon et al. 2020).

Based on the prior conservation activities, in 2015, the re-introduction programme for the Saker Falcon in Bulgaria was initiated with the objective to upgrade on the previously-conducted work by using a standardised and unified approach of releasing a set number of birds over a certain period of time utilising the hacking method. This establishment phase referred to the period in which the population is susceptible to threats that will disappear if the population survives this stage (Armstrong and Seddon 2008). From 2015, harness mounted transmitters were not used on the Saker Falcons as a study had shown they have a negative impact on the survival and breeding behaviour of the species (Dixon et al. 2016), which conflicts with the primary aim of maximising the survival of released individuals.

## Material and methods

### Captive breeding

Creating a captive-breeding group of Saker Falcons of known European origin from the Pannonian population was chosen over translocation of wild-sourced birds as the more practical option for obtaining birds to be released in the wild in Bulgaria (Dixon et al. 2020). The birds were imported from Austria, Hungary, Germany, Slovakia and Poland. The Green Balkans Wildlife Rehabilitation and Breeding Centre (WRBC) in Stara Zagora was equipped for facilitating captive breeding. Ten breeding aviaries and two stock cages for juvenile falcons were constructed and equipped with internal surveillance cameras. From 2015, a 5-year breeding and release programme was initiated. Funding was withdrawn between 2017-2019 and the breeding programme was maintained, but releases were not at the expected scale. In 2020, the programme was restarted, again with a 5-year plan.

### Hacking method

**Figure 1.**
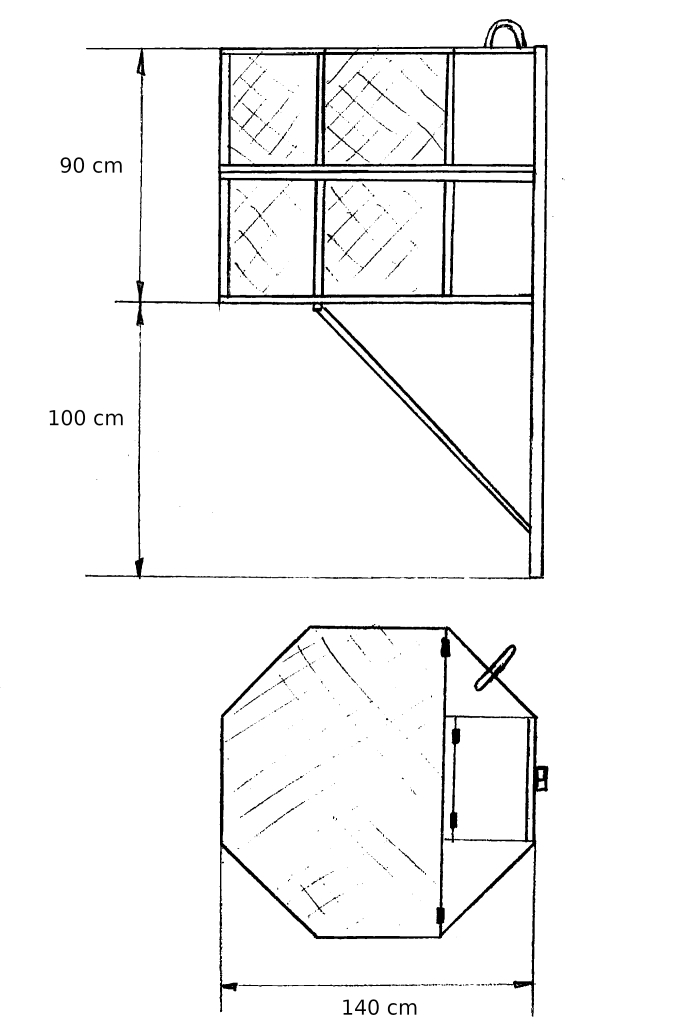
Saker Falcon hack cage from the side and top

**Figure 2.**
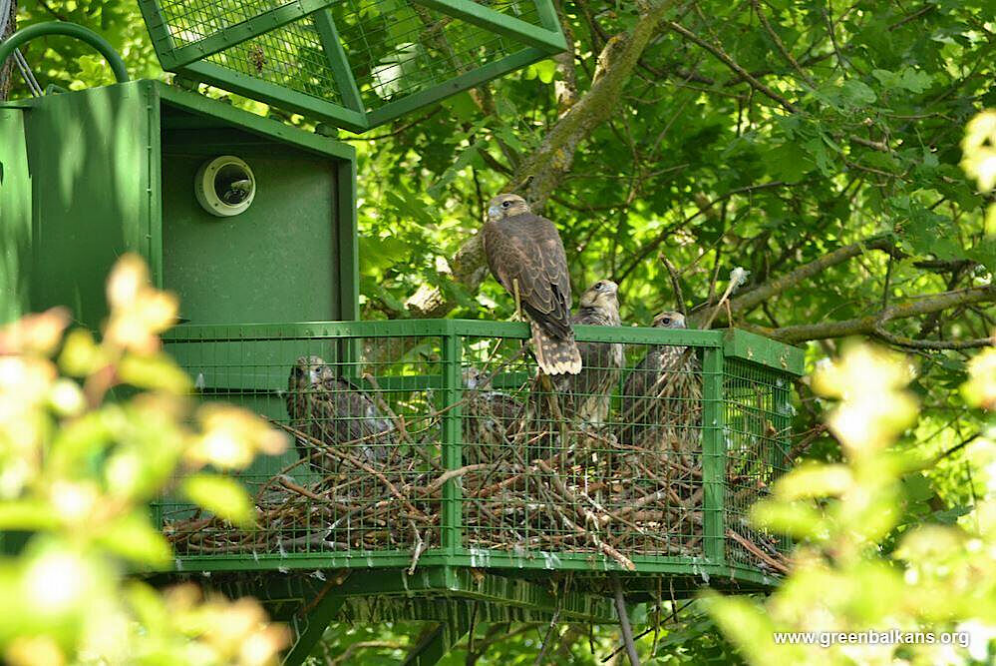
Photograph of an opened hack cage

Four аdaptation аviaries (or hacks), have been constructed and installed on oak trees at a height of 7-8.5 m and 0.7-2.8 km distant from each other, for the purposes of re-introduction in the territory chosen for release in Stara Zagora Province. The hack cages were octagonal steel structures, 150 cm in diameter and with a height of 90 cm. Five of the eight walls were of steel mesh, as was the roof. A hinge mechanism allowed the top part of the cage to be raised in order to open the hack. The cages were based on a model used for a Peregrine Falcon restoration project in Poland (Sielicki and Sielicki 2009) (Fig. 1Fig. 2).

This construction was suitable to be placed on old-growth trees; however, it is too heavy for installation on younger ones. As old-growth trees are rare in the area of release, the hack cages should be modified in case they need to be moved or if more are to be installed. The rails supporting the bottom could be cut out and additional hooks placed for mounting.

The hacks were equipped with surveillance cameras looking in and down on the cage. Up to 90 m away, feeding tables were set up in direct view of each aviary. Following a veterinary health check, during which each Saker Falcon was equipped with a microchip and a set of identification rings, the birds were transferred from the breeding facility to the hack sites at an age coinciding with their ability to fledge - around 30 days-old. They were placed in the cages in groups of three to five and kept within them for the first 10 days to avoid risk of predation. During that period, they were monitored via the internal surveillance cameras.

The falcons were fed twice or, in particularly hot summers, three times a day via a pulley mechanism in order to minimise human association with the feeding process. In addition, in the first ten days, food was placed three times - every three days, on the feeding table so the hacked birds would see it from the cage. From the opening of the hack, food was placed daily on the feeding table as well as in the hack. For the first 10 days from opening, it was placed twice a day in the hack and, only in the mornings, larger and more noticeable pieces were put on the table; for the second 10 days - twice a day on the table and once in the hack where more unappealing food was put to encourage the young birds to spend more time outside the hack cage. Six days after that, three times or every two days and for another six in which two times (every three days) more unappetising food was placed in the hack and regular provisions were provided twice a day every day at the feeding table. The cage’s top was closed 32 days after opening the hack and food was set from then on twice a day at the feeding table only. This was done until the end of July. In August, the feeding was dependent on whether Saker Falcons were still present in the release area - on average, once a day for 20 days, food was placed on the feeding table in the afternoons after 5.00 pm.

During the daily observations, the presence of each individual Saker Falcon was noted, its behaviour, attendance to the hack and feeding table and hunting attempts closely monitored as well as noting fledging date and date of individual birds’ last sighting in the release area. The field activities were conducted for four months each season. In 2015 and 2016, full-day monitoring was conducted for 54 and 74 days, respectively, from the opening of the hack with the first group of Saker Falcons. For the period 2017-2019, monitoring was not done full-time. In 2020, systematic full-day observations were again conducted for 74 days after the opening of the first hack cage.

### Statistical analysis

The data received was processed with IBM SPSS Statistics (SPSS-Inc. 2019, SPSS Reference Guide 26 SPSS, Chicago, USA) using descriptive statistics with frequency distribution tables. Correlation analysis (Pearson Correlation Coefficient) and Student t-test (t-test for independent samples, Levene's test p < 0.05) were used. The results afterwards were presented on diagrams (Excel, Windows 10).

### Population model

The model was created using RAMAS GIS 4.0, RAMAS Metapop 4.0 programme (Akçakaya and Root 2002) - a tool for developing population viability analysis (PVA) models of different complexity levels using built-in submodels. It was constructed for the purpose of estimating the growth of a new viable population of Saker Falcons in Bulgaria. The model described a single population consisting of individuals of different age and sex classes. The dynamics of this population were determined by age and sex class-specific survival rates and the initial number of individuals in each sex and age class.

For the purpose of modelling, three life stages of the Saker Falcons were determined: juvenile (< 1 year old), sub-adult (2 years old) and adult (> 3 years old). We accepted the first breeding to be at 3 years of age, the adult breeding rate to be 90%, the nest productivity - 2.6 juveniles per breeding pair and that the population would have an emigration rate of 10%. We have not built in any sex differences in the breeding age for the preliminary model. In 2020, the breeding output did not meet the minimum threshold for the restoration programme; for this reason, the modelling was re-adjusted to accommodate the next four years of the establishment phase, which would be considered complete when the planned threshold of a minimum of six Saker Falcon pairs breeding in the wild in Bulgaria is reached. The model was based on the planned release of 100 Saker Falcons in five consecutive years. The simulations were initialised with no adult individuals, only juveniles were introduced. The minimum number of released juvenile birds per year was planned as follows: in 2020 - 12 birds, 2021 - 18, 2022 - 20, 2023 - 25, 2024 - 25.

## Results

### Breeding performance

During 2015 and 2020, 70% (5-9) of the 9 to 11 breeding pairs maintained in the captivity have laid eggs. The average clutch size and brood size was 5.08 ± 0.22 eggs and 2.33 ± 0.46 nestlings. The ratio of couples raising at least one chick compared to the total number of breeding pairs for the study period was 0.69 ± 0.11. The average number of fledglings coincided with the breeding success (number of fledglings related to the number of pairs registered to have laid eggs). The number of fledged birds related to the number of pairs raising at least one chick was 2.77 ± 0.31 (Table 1).

### Hacking

For the period 2015-2020, 80 Saker Falcons were released in total via the hacking method from four aviaries near the town of Stara Zagora. Female birds represented 27 of them, the rest (53) were male. Sixteen individuals have been sourced from elsewhere, the rest (64) have been bred and hatched at the WRBC (Table 2). The lower number of released Saker Falcons in 2017-2019, compared to the first two years, was due to withdrawn funding and in 2020 - due to low breeding productivity of the captive breeding pairs, a number of which were of old age and infertile.

The compiled data of the 80 released Saker Falcons showed the birds were hacked, on average, at 32 days of age, with no significant difference between male and female birds (31.74 ± 0.84 and 32.34 ± 0.40, P = 0.47). Greater variation was found in the average weight prior to hacking - for males, it was 820.37 ± 9.28 g, while for females, it was 1026.89 ± 14.14 g. (P = 0.00). Similarly, regarding sex, there was a difference found in the average length of the tarsus - 28.78 ± 0.46 mm for males vs. 31.06 ± 0.35 mm for females respectively (P = 0.00). The average age at which Saker Falcons were when the cages were opened, was estimated at 42 days (41.30 ± 0.86 for males and 42.21 ± 0.47 for females (P = 0.00). Male birds were recorded to be 48.26 ± 1.69 days-old on average when first observed to be fledged from the adaptation aviary and females - 50.17 ± 1.04 days-old (P = 0.32). The maximum age at which Saker Falcons have been positively identified to do so (at 76 days-old) was due to irregular monitoring at the time. On average, the birds were observed to stay in the hack area until 82 days-old (males at 82.00 ± 3.09 and females until 81.53 ± 1.96, P = 0.89) (Table 3).

Regarding the retention in the release area, observation records in 2018 confirmed the presence of at least one pair currently breeding in the wild in the country, in 2018 and 2019, formed by the two released Saker Falcons in 2015. From 2020, it was composed of the same male with a changed female of the birds released in 2016 as part of the re-introduction programme. The three Saker Falcons of the breeding pair have been systematically observed in the hack area, until dispersing at 75, 77 and 87-days-old. Retention in the proximity of 100 km to the release site was confirmed also in early 2021 for a Saker Falcon released in 2020, as for other Sakers in previous years (2018-2020). All of the returned birds that were identified were ones which had spent a significant amount of time in the release site.

### Modelled population growth

**Figure 3.**
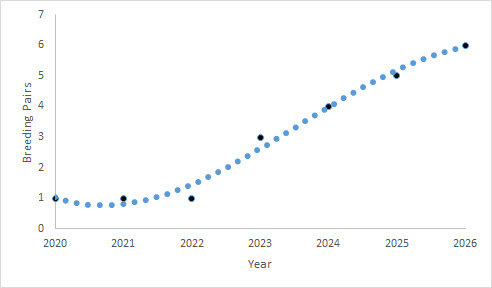
Preliminary model of Saker Falcon population growth for 2020-2026.

A positive population growth was observed - six pairs were expected to be formed by 2026, which indicated the establishment of a self-sustaining population and a successful re-introduction (Fig. 3).

## Discussion

Captive breeding and consecutive releasing in the wild with the aim of species preservation and restoration has proven as a successful method since the mid-20^th^ century (Cade et al. 1988). It has played an important role in the re-introduction of the Peregrine Falcon in Europe (Lindberg and Sjoberg 2009).

A re-introduction programme for the Saker Falcon was initiated in 2015 in Bulgaria. For the studied period, the average clutch size was 5.08 ± 0.22 (from 5-9 breeding pairs), which appeared to be higher than the average for wild Saker Falcons in Ukraine - 4 (Karyakin et al. 2011) and Hungary - 3.6 (Gál et al. 2007). It was also higher than the clutch size of the Lesser Kestrel (4.5-4.9) also breeding in captivity at the WRBC (Gradev et al. 2016). A considerable difference was observed between the clutch size and brood size (2.33 ± 0.46) of the breeding Sakers. The reason for such dissimilarity in the values might be attributed to the old age of some of the Saker Falcons and specifically to seven of the breeding pairs which, during the studied period, all had laid a clutch and which, during ovoscopy, was found to be infertile and taken from the nest, in turn making them lay a second clutch. This, unfortunately was also found to be infertile. The brood size value was thus much closer to the average recorded in the wild - 2.83 ± 0.78 (Karyakin et al. 2011).

Data from morphometrical and mass measurements of the hacked birds showed that the mass of the males was equivalent to 79% of the mass of the females, which is expected by sexual dimorphism of the species. These results are consistent with the findings of Ferguson-Lees and Christie (2001) who reported the size of the male to be roughly equivalent to 72% of that of the female. The tarsal width was also measured with regards to sex determination. These results are related to assessing the health of the birds prior to their hacking and are essential in establishing the sex of the juvenile Saker Falcons (Kenward et al. 2001).

The 80 Saker Falcons released over 2015-2020 in Bulgaria were recorded to have fledged from the hack sites on average between 40-50 days-old, an age comparable to that of wild birds from natural nests (Baumgart 1991, Orta et al. 2020). During the PFDP, at natural nests, it was reported by Newton (1979) and Bustamante (1993) that fledglings continued their development depending on some form of parental care. During this period, they were imprinting on the natal area learning to hunt by themselves. Comparing the average age of dispersal (age at last sighting) of the hacked Saker Falcons in Bulgaria during 2015-2020, which was estimated to be 82 days, we found it exceeded the age range of dispersal reported for three captive bred Saker Falcons hacked in Austria (62–65 days old) (Gamauf and Dosedel 2012). In Central Europe, the PFDP of wild Saker Falcons was considered to average one and a half months after fledging (Prommer et al. 2012); in Ukraine, the same period lasted 44 days on average (range 34–64 days; Prommer et al. 2014). The 82-days dispersal age also appeared to be more than the average of the Saker Falcons released during the pilot study, which corresponded with the data above (Dixon et al. 2020).

The significance of the PFDP was underlined by reports relating juvenile survival to the length of time spent in the release area after fledging, considering the survival prospects of satellite-tagged Sakers to be “minimal” for juveniles that left their natal area very early (Prommer et al. 2012). Similar observations were reported for the hacked Peregrine Falcons which showed reduced survival in cases of dispersal prior to the optimal 3-4 week PFDP (Dzialak et al. 2007). These facts clearly identified the necessity and significance of hacksite management activities, especially developed and implemented to retain birds in the natal area and thus to increase their chance for surviving. Part of these activities, which could assist in retaining the birds in the release area for a time closer to the natural PFDP, appeared to be the regular provision of food at the hack cages and nearby feeding stations (Sherrod 1983). This approach was used successfully throughout the studied period of the Saker Falcon re-introduction programme and fledglings were successfully retained in the vicinity of the hacks by food provisioning twice a day at the aviaries and feeding tables for up to three months.

Final outputs of the Saker Falcon re-introduction programme appeared to be dependent both on the number of released individuals and their survival rate after fledging. Due to the lower breeding output and unachieved minimum of released birds in 2020, as the programme was restarted, the population model was adjusted in terms of the number of Saker Falcons to be released each season. In this regard, in order to calculate population growth in our study, we assumed juvenile survival rates of 35%, sub-adult survival rates of 60% and adult survival rates of 80%. For comparison, Ragyov et al. (2009) created possible scenarios of new population establishment in their feasibility study citing that juvenile survival of 40% was provisionally taken from 10 Hungarian juveniles that were fitted with satellite transmitters. For the subadult/adult life stage, survival rate of 82% was reported from radio-tracking and DNA studies in Kazakhstan (Wink et al. 1999, Kenward et al. 2007). The current establishment phase and the re-introduction programme will be considered successful as the goal of releasing 100 Saker Falcons over a five ­year period from 2020 is met and six pairs are breeding in the wild in the country, with the purpose of restoring the Saker Falcon population in the southern Balkans and facilitating gene flow amongst fragmented populations from Central Europe to Kazakhstan. In doing so, we will develop transferable skills and knowledge that can be used for captive breeding­ and ­release programmes elsewhere. The success of the re-introduction programme depends on the released Sakers breeding in the agricultural landscape of central Bulgaria, thereby establishing a nucleus for the development of a sustainable breeding population.

## Conclusion

The positive experience of two Saker Falcons released beforehand in 2015 near Stara Zagora within the re-introduction programme, which were, in 2018 and 2019, confirmed to be effectively breeding in the wild, proved that hacking could result in Sakers surviving in the wild until maturity and that they could return to the region of their release and breed successfully. This was reinforced by the change of the female bird in 2020 with a Saker Falcon released in 2016 and of a successful breeding season for the new pair. Along with sightings of previously released birds in the region of re-introduction between 2018-2020, a sighting was reported in early 2021 of a Saker Falcon newly-released in 2020, recorded 100 km from the release site. All the returned birds have spent a significant amount of time in the hack area prior to dispersal. These results indicated the release of captive-bred Saker Falcons via hacking appeared to be an appropriate option for restoring the Saker Falcons in a part of their former breeding range. The data obtained, so far, point to the importance of hacksite management which, together with an improved breeding output, can contribute to more quickly achieving the objectives of the re-introduction project. In 2020, the programme was restarted on the basis of planned releases of 100 Saker Falcons in total over five years, to meet the minimum threshold for a modelled population. The establishment phase would be considered complete when six breeding pairs of Saker Falcons are registered in the wild in Bulgaria. The expected result of the programme is to successfully establish a self-sustaining population of the species in Bulgaria. Future benefits are linked with expectations that this founding population will attract further breeding recruits from neighbouring regions in Central and Eastern Europe, thus facilitating gene flow amongst existing fragmented populations. Furthermore, the re-­establishment of this iconic species has profound implications for conservation in Bulgaria in terms of public awareness of species conservation and as an indicator of wider environmental issues.

## Figures and Tables

**Table 1.** Saker Falcon breeding performance for the period 2015-2020

	Mean ± Std. Err.	Range (Min-Max)
Clutch size	**5.08** ± 0.22	1.50 (4.60-6.10)
Brood size	**2.33** ± 0.46	3.00 (1.20-4.20)
Success Rate	**0.69** ± 0.11	0.60 (0.40-1.00)
Productivity	**1.95** ± 0.45	2.80 (1.00-3.80)
Breeding success	**1.95** ± 0.45	2.80 (1.00-3.80)
Fledging success	**2.77** ± 0.31	1.80 (2.00-3.80)

**Table 2.** Number (n) of released Saker Falcons by hacking for the period 2015-2020.

Year	♂/n	♀/n	WRBC/n	Imported/n	Total Released/n
2015	8	11	19	0	**19**
2016	8	11	7	12	**19**
2017	6	10	14	2	**16**
2018	0	6	6	0	**6**
2019	1	7	8	0	**8**
2020	4	8	10	2	**12**
Total/n	**27**	**53**	**64**	**16**	**80**

**Table 3.** Post-fledging dependence period data of Saker Falcons, 2015-2020.

Sex		Age Hacked/ days	Weight Hacked/ g	Tarsus/mm	Age Hack opened	Post-fledging dependence period	
						Age First sighting after fledging	Age Last sighting
♂	Mean ± SE.	**31.74 ± 0.84**	**820.37 ± 9.28**	**28.78 ± 0.46**	**41.30 ± 0.86**	**48.26 ± 1.69**	**82.00 ± 3.09**
	Min.-Max.	27.00-49.00	700.00-890.00	22.00-35.00	37.00-59.00	38.00-76.00	59.00-120.00
♀	Mean ± SE	**32.34 ± 0.40**	**1026.89 ± 14.14**	**31.06 ± 0.35**	**42.21 ± 0.47**	**50.17 ± 1.04**	**81.53 ± 1.96**
	Min.-Max.	27.00-40.00	880.00-1235.00	21.00-37.00	36.00-51.00	42.00-76.00	43.00-110.00
Total	N	80	80	80	80	80	70
	t-value	-0.73	-9.87	-3.85	-1.01	-1.01	0.13
	p	0.47	0	0	0.32	0.32	0.89
